# Insights into the influence of cell concentration in design and development of microbially induced calcium carbonate precipitation (MICP) process

**DOI:** 10.1371/journal.pone.0254536

**Published:** 2021-07-12

**Authors:** Raja Murugan, G. K. Suraishkumar, Abhijit Mukherjee, Navdeep K. Dhami

**Affiliations:** 1 Bhupat and Jyoti Mehta School of Biosciences, Indian Institute of Technology Madras, Chennai, India; 2 School of Civil and Mechanical Engineering, Curtin University, Perth, Western Australia, Australia; University of Naples Federico II, ITALY

## Abstract

Microbially induced calcium carbonate precipitation (MICP) process utilising the biogeochemical reactions for low energy cementation has recently emerged as a potential technology for numerous engineering applications. The design and development of an efficient MICP process depends upon several physicochemical and biological variables; amongst which the initial bacterial cell concentration is a major factor. The goal of this study is to assess the impact of initial bacterial cell concentration on ureolysis and carbonate precipitation kinetics along with its influence on the calcium carbonate crystal properties; as all these factors determine the efficacy of this process for specific engineering applications. We have also investigated the role of subsequent cell recharge in calcium carbonate precipitation kinetics for the first time. Experimental results showed that the kinetics of ureolysis and calcium carbonate precipitation are well-fitted by an exponential logistic equation for cell concentrations between optical density range of 0.1 OD to 0.4 OD. This equation is highly applicable for designing the optimal processes for microbially cemented soil stabilization applications using native or augmented bacterial cultures. Multiple recharge kinetics study revealed that the addition of fresh bacterial cells is an essential step to keep the fast rate of precipitation, as desirable in certain applications. Our results of calcium carbonate crystal morphology and mineralogy via scanning electron micrography, energy dispersive X-ray spectroscopy and X-ray diffraction analysis exhibited a notable impact of cell number and extracellular urease concentration on the properties of carbonate crystals. Lower cell numbers led to formation of larger crystals compared to high cell numbers and these crystals transform from vaterite phase to the calcite phase over time. This study has demonstrated the significance of kinetic models for designing large-scale MICP applications.

## 1. Introduction

Microbially induced calcium carbonate precipitation (MICP) has recently emerged as a potential technology for improving the engineering properties of different construction materials [1–4]. The process is based upon harnessing the metabolic activity of microorganisms which lead to changes in their microenvironment and cause precipitation of calcium carbonate minerals such as limestone [5]. Amongst the different metabolic pathways via which microorganisms lead to precipitation of calcium carbonate in natural environments, MICP via ureolytic pathway has been the most widely explored for application purposes [6]. This pathway offers the benefits of high efficacy and straightforwardness [5–7] and has been successfully utilised for improving the mechanical properties of different granular materials including soil and cement [8, 9].

During MICP process, bacterial cells producing the enzyme urease hydrolyse urea to form ammonium and carbonate ions; which then react with the soluble Ca^2+^ ions to form calcium carbonate [3, 5] (Eqs 1 and 2).


$$Co(NH_{2}{)}_{2}\;+\;2\;H_{2}O\;\to \;HCO_{3}^{2-}\;+\;2NH_{4\;}^{+}+\;{OH}^{-}$$ (1)

$${Ca^{2+}\;+\;CO}_{3}^{2-\;}+bacteria\;\to bacteria-\;CaCO_{3}↓$$ (2)

From the stoichiometry (Eqs 1 and 2), MICP is chiefly divided into two major steps: urea hydrolysis and CaCO_3_ precipitation. It is evident that the equimolar concentration of calcium and urea is required to achieve the maximum amount of calcium carbonate in the system. Direct utilisation of urease enzyme for inducing calcium carbonate precipitation has also been attempted in a few studies [10–12]. Due to the susceptibility of enzymes to environmental conditions as well as high costs, MICP has been the preferred mode of application for engineering applications [10, 11, 13, 14]. It has also been recorded that microbially induced carbonate crystals are relatively larger than the crystals induced by an equivalent amount of enzyme offering a better alternative [12].

The overall efficacy of the MICP process is dependent upon a number of physicochemical and biological factors including temperature, pH, concentration of nutrients, cementation reagents and most importantly on the concentration of bacterial cells and urease enzyme [15–20]. The concentration of bacterial cells is proportional to the availability of nucleation sites for carbonate deposition and total urease production [16]. Cell concentration has a major impact on the overall efficacy and kinetics of ureolysis as well as the calcium carbonate precipitation process. The rate and kinetics of calcium carbonate precipitation, in turn, affect not only the quantity of calcium carbonates but also the morphology and quality of precipitated crystals. In nature, calcium carbonate exists in six polymorphs including calcite, aragonite, vaterite, CaCO_3_ monohydrate, CaCO_3_ hexahydrate to amorphous CaCO_3_ [15, 21]. It has been recorded earlier that the shape and size of CaCO_3_ crystals changes during the precipitation process. The crystal patterns and polymorphs have a significant impact on the strength and stiffness of the cemented substrate [22, 23]. Amongst all the polymorphs, calcite is the most stable and desirable polymorph of calcium carbonate for engineering applications [24, 25]. Previous studies found that bacterial strains with lower urease activity slowed down the precipitation process and yielded high-quality calcite indicating the significant influence of urease activity on crystal property [26–28]. In another study, it was recorded that higher urease activity of *Bacillus sp*. could result in higher CaCO_3_ content while lower urease activity resulted in generating 10 times bigger crystals [29]. All these factors will impact calcium carbonate crystal behaviour and have a direct impact on the particle bond failure mechanism determining the efficacy of soil cementation. To design an effective MICP process for engineering applications, it is therefore mandatory to understand the influence of cell concentration on the kinetics of ureolysis and calcium carbonate precipitation under application—related conditions.

Few studies have been conducted on exploring the effect of bacterial cell concentration on the kinetics of urea hydrolysis and CaCO_3_ precipitation earlier [12, 30–32] and it has been found that changing the initial inoculum size from optical density (OD) 0.03 to 0.07 leads to a 10-fold change in the rate constant of urea hydrolysis [31]. Linear relationship between the initial ureolysis rate and cell concentration in the absence of calcium in the medium has been reported earlier [32]. Improvement in the maximum kinetic constant value of calcium carbonate precipitation from 0.027 h^-1^ to 0.048 h^-1^ by increasing the initial cell concentration 10-fold has been seen by other researchers [12]. Although these studies have recognized the positive impact of cell concentration on the rate of MICP process, detailed insights on their influence at higher doses are required to ensure the efficacy and speed of the process for engineering applications [32]; especially in the concentration used in field-scale applications.

The most widely used method for utilisation of MICP technology for engineering applications in soils comprises of two phases. In the first phase, the harvested bacterial cells are mixed with the substrate soils followed by pumping of cementation reagents with urea—CaCl_2_ (0.5M) in the second phase [33]. Very limited information is available on its precipitation kinetics and its effect on the carbonate polymorph under the conditions applicable to soil applications. It is therefore imperative to study the process kinetics under the same conditions in detail. In addition to that, it will be vital to have a mathematical model equation to relate the kinetic parameters of MICP with the initial cell concentration for improving the efficacy of the process.

In real—time application of MICP, injections of bacterial cells and cementation media reagents are repeated multiple times (recharges) in order to fill up the substrate pores in soils and achieve the required amount of cementation [3, 8]. The studies utilising multiple recharges of MICP treatment have limited information on how different recharges of bacterial cells influence the speed of carbonate precipitation over a period of time. Also, the optimal dosage of cells and cementation reagents for achieving the desired cementation in a specific period needs to be known.

Therefore, this study aims to get detailed insights on the influence of cell concentration on precipitation kinetics for design and development of field scale MICP applications. In addition to the improvement of the kinetics of calcium carbonate precipitation, it would be significant to have a mathematical model equation to relate the kinetic parameters involved in MICP with varying cell concentrations. Along with this, the effect of cell concentration on carbonate crystal morphology will aid in design and development of the MICP process for soil applications requiring different levels of cementation. The major objectives of this study are therefore to:

a) investigate the influence of initial cell concentration on the kinetics of the urea hydrolysis and CaCO_3_ precipitation b) check the impact of initial cell concentration on the calcium carbonate crystal morphology and phase c) develop a mathematical equation between the kinetic parameters associated with MICP and cell concentration d) investigate the efficacy of bacterial cells in cementation under multiple recharge conditions.

## 2. Materials and methods

### 2.1 Inoculum preparation, media composition, measurement of OD

The strain used in the present study is *Sporosarcina pasteurii* (ATCC 11859). The culture was prepared using the medium (ATCC 1376) containing yeast extract (20 g/L), ammonium sulphate (10 g/L), 0.13 M tris base (pH 9) [5]. The components of the media were prepared separately and mixed after autoclaving. The culture was centrifuged, and the pellet was dissolved in 0.85% of sodium chloride solution to measure its optical density (OD) using a spectrophotometer @600 nm (Thermo scientific, Genesis 10S), where 0.85% sodium chloride solution was used as a blank.

The cementation medium used in this study contains 2 g/L of yeast extract, 0.5 M urea, and CaCl_2_·2H_2_O [33]. 65 mL of deionized water containing 2 g of yeast extract was prepared and pH was adjusted to 8.0 with 1N NaOH solution and autoclaved separately. Then 5 M and 2 M filter-sterilized urea and CaCl_2_·2H_2_O stock solutions were prepared. From the stock solution, 10 mL of urea, and 25 mL CaCl_2_·2H_2_O were added into the autoclaved yeast extract solution to achieve a final concentration of 0.5 M urea and CaCl_2_·2H_2_O. The overnight culture of *Sporosarcina pasteurii* was centrifuged, and the pellet was inoculated into the cementation medium to achieve different initial cell concentrations (0.1 OD to 0.5 OD).

The relationship between colony-forming units/mL and OD@600 nm was plotted by performing the colony-forming unit assay in the growth medium containing 1.5% agar. To plot the relationship between dry cell weight and OD@600 nm, the known concentrations of cells were dried at 70 °C for overnight. After the drying, it was weighed using a weighing balance.

### 2.2 Study design

An experimental design to study the influence of bacterial cell concentration on the kinetics of ureolysis, calcium carbonate precipitation and morphology of the CaCO_3_ precipitate has been shown in Table 1.

**Table 1:** Experimental design to study the influence of bacterial cell concentration on the kinetics and morphology of the CaCO_3_ precipitate.

Initial cell concentration OD @600 nm	Cell number x 10^7^ CFU/mL	Dry cell weight g/L	Measured parameters	Morphology and phase analysis of calcium carbonate
Control (No cells)	0	0	The concentration of soluble calcium, urea and ammonium ions, pH and dry weight of insoluble precipitate	Scanning Electron Microscopy–Energy Dispersive spectroscopy. X-Ray Powder Diffraction
0.1	4.5	0.04		
0.2	9.0	0.08		
0.3	13.5	0.12		
0.4	18.0	0.16		
0.5	23.5	0.2		

Kinetics of calcium carbonate precipitation after the second recharge of cementation solution with and without the addition of fresh cells was monitored till complete precipitation of CaCO_3_. In the first recharge, 6 flasks were inoculated with 0.4 OD as initial cell concentration into the cementation medium. After the 6^th^ hour, the precipitates from all the flasks were centrifuged (5000 rpm for 10 min) along with bacterial cells. In the second recharge, 2 sets (A and B) of three flasks containing cementation medium were used. Each flask of set A was inoculated with the pellet from the first recharge. Each flask of set B was inoculated with the pellet from the first recharge as well as 0.4 OD of fresh overnight grown bacteria. An experimental design to study the influence of cells on the kinetics of CaCO_3_ precipitation after the subsequent recharge of cementation medium has shown in Table 2.

**Table 2:** Experimental design to study the influence of cells on the kinetics of CaCO_3_ precipitation after the subsequent recharge of cementation medium.

First recharge	Second recharge	
	Set A	Set B
Cementation medium containing 0.4 OD of overnight grown bacteria.	Cementation medium containing precipitate from the first recharge.	Cementation medium containing precipitate from first recharge and 0.4 OD of overnight grown bacteria.

The samples were taken from the cementation medium centrifuged at 3000g for 10 minutes and the supernatant was used to measure concentrations of urea, calcium ions, ammonium ions, and pH. The pellet was washed twice with distilled water and used to measure the dry weight of the insoluble precipitate.

### 2.3 Measurement of urea concentration

The urea concentration of the samples was measured using the Dimethylaminobenzaldehyde (DMAB) method [34]. In this study, 50 μL of the sample was added to the 50 μL of a 12% trichloroacetic acid solution. This was followed by the addition of 100 μL of DMAB reagent (containing 1.6% of DMAB in concentrated hydrochloric acid containing 10% (v/v) ethyl alcohol). The mixture was incubated for 5 minutes at room temperature. The optical density of the yellow colour obtained in the mixture was measured at 425 nm using a plate reader. The standard was plotted between optical density at 425 nm vs urea concentration (0–125 mM). The slope value obtained from the plot was used to measure the urea concentration of the samples.

### 2.4 Measurement of ammonium ions concentration

The ammonium ion concentration of the sample was measured using the Phenol-hypochlorite assay [35]. 130 μL of diluted sample was added to 35 μL of the phenol-nitroprusside reagent and 35 μL of alkaline hypochlorite reagent. The mixture was incubated for 30 minutes at 37 °C. The optical density was measured using a plate reader (Thermofisher Scientific). The standard was plotted between optical density at 626 nm vs ammonium concentration (0–100 μM). The slope value obtained from the plot was used to measure the ammonium ions concentration of the samples.

### 2.5 Measurement of soluble calcium

The complexometric titration method was used to estimate the soluble concentration of Ca^2+^ in the supernatant [36]. 40 μL of supernatant was diluted into 10 mL and 400 μL of 1N NaOH was added to the solution to raise the pH. After that, a few drops of hydroxy naphthol blue disodium salt (1% W/V) solution was added as an indicator. The titration was performed against 1 mM EDTA disodium salt solution until the colour changes from pink to blue, and the endpoints were noted. In this study, the standard solutions of 0 to 2 mM CaCl_2_ were prepared and titrated against 1 mM titrant solution. The endpoints of the standard solutions were noted. A graph was plotted between the concentrations of CaCl_2_ solution vs. the volume of 1 mM EDTA required to reach the endpoint. An unknown sample concentration was found from the slope of the plot.

### 2.6 Measurement of pH and CaCO_3_

The pH of the supernatant solution was measured using a pH meter (Thermo scientific, Orion star, A211). To measure CaCO_3_ the insoluble precipitate of the sample was dried at 70 °C for overnight. The dry weight of the precipitate was measured using the weighing balance.

### 2.7 SEM-EDS and XRD analysis

30 mL of culture medium was taken, and the pellet was collected through centrifugation. The pellet was washed with distilled water and dried at 37 °C. The dried pellet was ground uniformly using mortar and pestle before its quality analysis by performing Scanning Electron Microscopy (SEM)–qualitative Energy Dispersive X-ray spectroscopy (EDS) analysis and X-ray powder Diffraction (XRD) methods.

#### 2.7.1 SEM-EDS—Morphology (size, shape) and composition

Scanning electron microscopy and qualitative EDS was used to analyse the morphology and elemental composition of the dried powder respectively. The variable pressure electron microscope (VP–SEM, Zeiss, EVO 40 -XVP, 2008) was used. The samples were coated on carbon-aluminium tape. Before the analysis, the surface of the samples was covered by carbon tape using a carbon evaporative coater (Creissington, 2080C, 2011). The images were taken at the beam intensity and voltage of 8.0 and 10 kV respectively with the working distance around 15 mm. Secondary electron imaging was used to obtain electron micrography. The size of the observed crystals was measured using IMAJEJ (1.8.0 172) software. The EDS spectrum was obtained at the accelerating voltage of 10 kV.

#### 2.7.2 Phase analysis of the crystals using X-ray powder diffraction method

The samples were resuspended in ethanol and deposited onto low-background holders. Data were collected with a Bruker D8 Advance diffractometer with Ni-filtered Cu Kα radiation (40 kV, 40 mA) over the range 7–120° 2θ, with a step size of 0.015°. The phase identification was carried out in Bruker EVA 5.2 using the COD database. Phase quantification was carried out in Topas Academic 7 by the Rietveld method. Crystal structures were taken from the COD.

### 2.8 Statistical analysis

All the experiments were run in triplicates. The solver function in excel (2016) was used for curve-fitting. One-way ANOVA was used to determine the statistical significance of the study. Student–t-test was used to find statistical significance between the groups using Graph Pad (Prism 7, 2016).

### 2.9 Calculation of kinetic parameters

**Fig 1:**
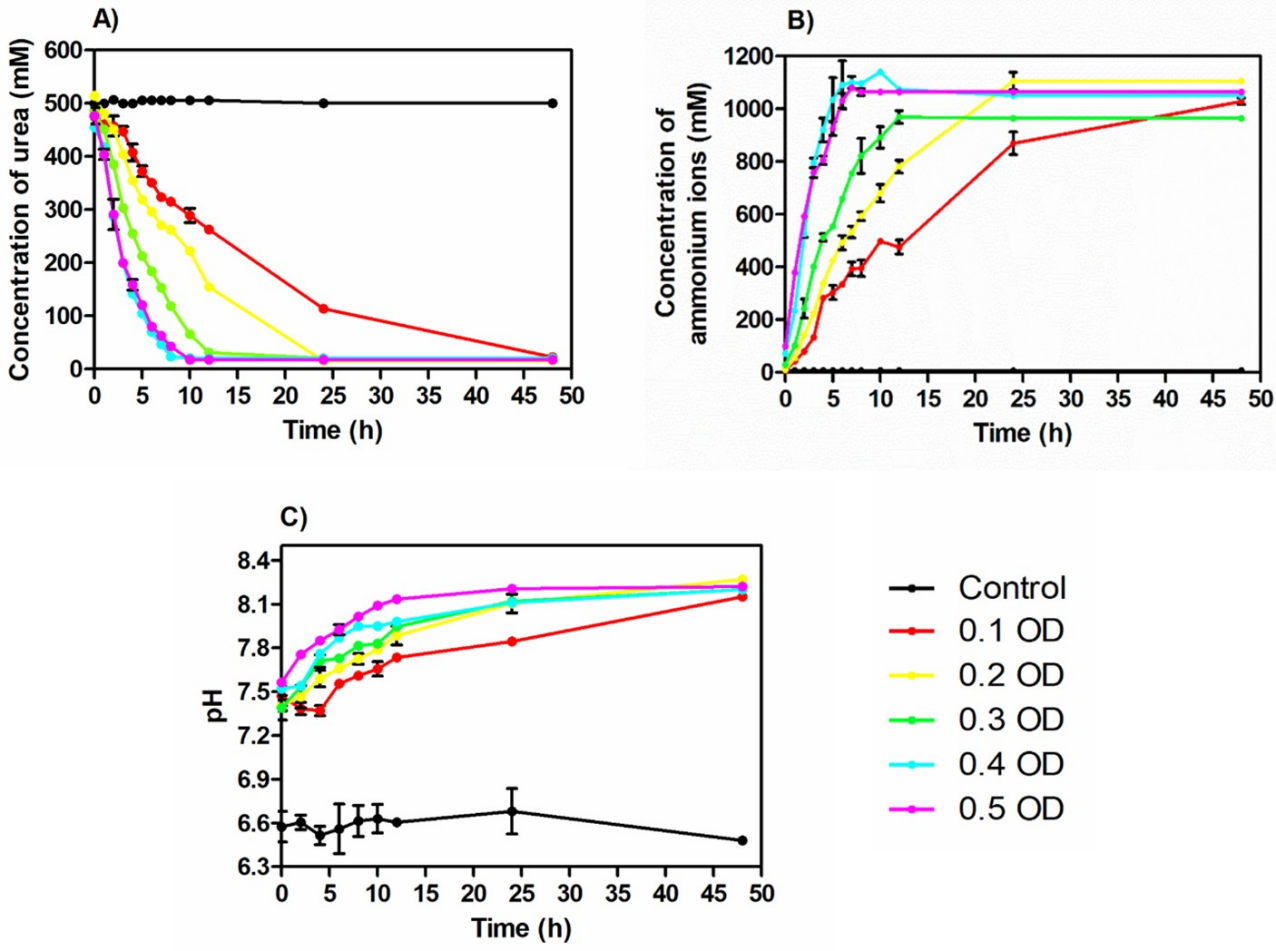
The concentration of urea (A), ammonium ions (B), and pH (C) over time.

**Fig 2:**
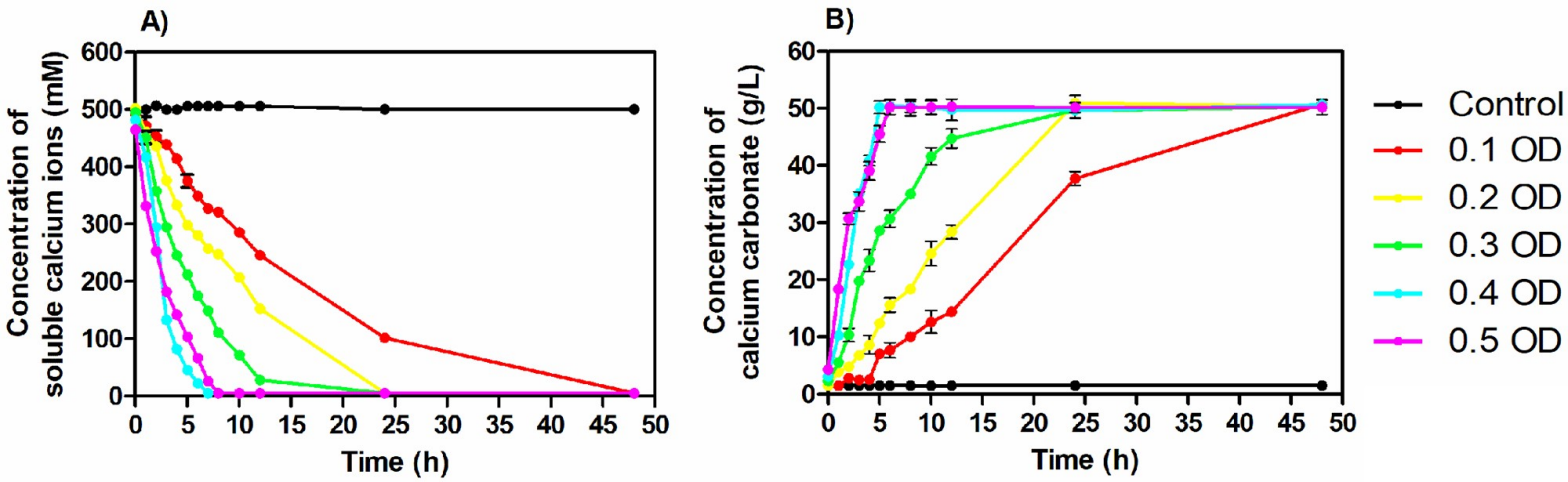
Concentration of soluble calcium ions (A) and calcium carbonate (B) over time.

The kinetic parameters associated with urea hydrolysis and CaCO_3_ precipitation calculated from the profiles of urea and soluble calcium, respectively (Figs 1A and 2A). The parameters are the maximum rate of urea hydrolysis (R_urea, Max_), the kinetic constant of urea hydrolysis (K_urea_), the maximum rate of calcium carbonate precipitation (R_cal, Max_), and the kinetic constant of calcium carbonate precipitation (K_cal_). The R_urea, Max,_ and R_cal, Max_ is a maximum slope of urea and calcium profile respectively. The K_urea_ and K_cal_ represent the urea and soluble calcium over time and their values were calculated by fitting in the logistic Eqs (3) and (4) respectively.

$$C_{urea\;}\left(t\right)\;=\;1000/(1+e^{k_{urea\;}t})$$ (3)

1000 in the numerator is an empirical constant (mM),

C_urea_ (t) = urea concentration (mM) at given time,

t = time (h) and

K_urea_ = kinetic constant of urea hydrolysis (h^-1^).

$$C_{cal\;}\left(t\right)\;=\;1000/(1+e^{K_{cal\;}t})$$ (4)

1000 in the numerator is an empirical constant (mM),

C_cal_ (t) = soluble calcium concentration (mM) at given time,

t = time (h) and,

K_cal_ = kinetic constant of calcium carbonate precipitation (h^-1^).

Further, the initial cell concentration in the given system is assumed to be the real-time cell concentration (X_0_ = X) due to the entrapment of bacterial cells within the carbonate crystals (it is very cumbersome to have an accurate picture of the cell numbers). The cell concentration normalized with K_urea_ and K_cal_ was used to compare the effect of cell concentration on the kinetics of urea hydrolysis and CaCO_3_ precipitation [31].

## 3. Results

### 3.1 Effect of cell concentration on urea hydrolysis

To understand the effect of initial cell concentration on urea hydrolysis, the concentration of urea was monitored over time. The urea concentration in the medium decreased with time in all the sets at varying rates (Fig 1A). It was seen that the medium inoculated with lower cell concentration (0.1 OD) took a much longer time for complete hydrolysis compared with the higher cell concentrations. In the case of sets with OD 0.4 and 0.5, immediate urea hydrolysis was recorded. Up to 50% of consumption was seen within 2–3 hours. Zero-order reaction rate was recorded in these sets in the initial phase of the process. It was found this is the phase when the hydrolysis rate of urea happens at its maximum. It is possible to calculate the zero-order rate constant from this phase for all the medium inoculated with 0.1 to 0.5 OD cell concentrations. Once the phase got over, the rate of hydrolysis started declining in all the groups and reaches zero at the end of the process.

From Fig 1B, the ammonium concentration in the medium was also recorded to increase over time in all the bacterial inoculated sets. The inverse trend of ammonium ions concentration was observed compared to the urea hydrolysis trend. It indicated the generation of ammonium ions due to urea hydrolysis. Similar to the urea concentration profile, in this case also a slower rate of increase in the ammonium concentration was observed in the initial time interval. The initial phase was found to decrease with increase in the initial cell concentration. In the medium inoculated with higher cell concentrations (0.4 and 0.5 OD), immediate production of ammonium ions was recorded. After this phase, the rate of change of ammonium concentration followed a zero-order reaction rate. The rate of generation of ammonium ions in the last phase started decreasing and reached its saturation concentration when no more urea was available for hydrolysis.

The pH of the medium was found to increase over time in all the bacterial inoculated sets. The pH values were observed mostly in between 7 to 8 during the process (Fig 1C). The cementation medium inoculated with a higher cell number showed a faster increase in the pH until it reached around 8.2.

### 3.2 Effect of cell concentration on calcium carbonate precipitation

To investigate the effect of bacterial cell concentration on the calcium carbonate precipitation, soluble calcium in the supernatant and insoluble precipitate (CaCO_3_) in the cementation medium were monitored throughout the process (Fig 2A and 2B). It was observed that the concentration of soluble calcium decreased over time (Fig 2A). The profile of soluble calcium followed a similar trend in all the groups inoculated with different initial cell concentrations.

The profiles followed a logistic curve. It was observed that the rate of soluble calcium concentration change was not uniform throughout the process. In this case, three different phases in the profile of soluble calcium ion concentration in the supernatant were observed (Fig 2A) as follows,

1. During the initial phase, the rate of calcium depletion was very slow in the low cell concentration range with OD ranging from 0.1–0.3. A lag of three hours, two hours and an hour was recorded before the start of decrease in concentration of Ca in case of cell concentrations with OD 0.1, 0.2 and 0.3. However, immediate reduction in the concentration of soluble Ca ions was recorded for high cell concentrations with OD 0.4 and 0.5. This demonstrated that the time taken by bacterial cells to cross the first phase/beginning of calcium consumption decreased with an increase in the initial cell concentration.
2. During the middle phase where maximum calcium depletion was recorded in all the cell concentration ranges, noticeable differences in the time taken for calcium depletion were again recorded. It varied from 3–7 hours for 0.1 OD, 2–6 hours for 0.2 OD, 1–5 hours for 0.3 OD and 0–4 hours for high cell concentrations with OD 0.4 and 0.5. The rate of change of soluble calcium during this phase followed a zero—order reaction. The maximum rate of soluble calcium depletion was calculated from this phase.
3. After the mid exponential phase with high rate of calcium reduction, a slow phase of depletion till 100% calcium is consumed was again recorded again and classified as the late phase. During this phase, the rate of calcium concentration depletion was slow in all the cell concentrations varying from 7 hours to 4 hours for OD 0.1–0.5. It was observed that during this phase, the bacterial cells were not able to precipitate calcium carbonate as quickly as in the middle phase.

The faster depletion of soluble calcium ions was found in the medium inoculated with higher cell concentration. In the case of 0.4 OD cell concentration, more than 90% of the soluble calcium ions (equivalent to 450 mM soluble calcium) were depleted within the first 6 hours.

The dry weight of the precipitate was also measured and plotted against time (Fig 2B). Similar to the soluble calcium profile, the profile of calcium carbonate precipitate also had three different phases. The rate of change of calcium carbonate precipitate concentration increased with increasing cell concentration. At the end of the process around 50 g/L of precipitate was observed in all the cementation medium inoculated with different cell concentrations which are stoichiometrically equal to the calcium supplied in the medium. In particular, the medium inoculated with 0.4 OD and 0.5 OD reached 50 g/L in around 6 hours while in case of low cell concentrations with OD 0.1, 0.2 and 0.3, more than 90% CaCO_3_ precipitation occurred after a time interval of 35, 20 and 12 hours.

### 3.3 Kinetics of ureolysis and calcium carbonate precipitation at different cell concentrations

**Fig 3:**
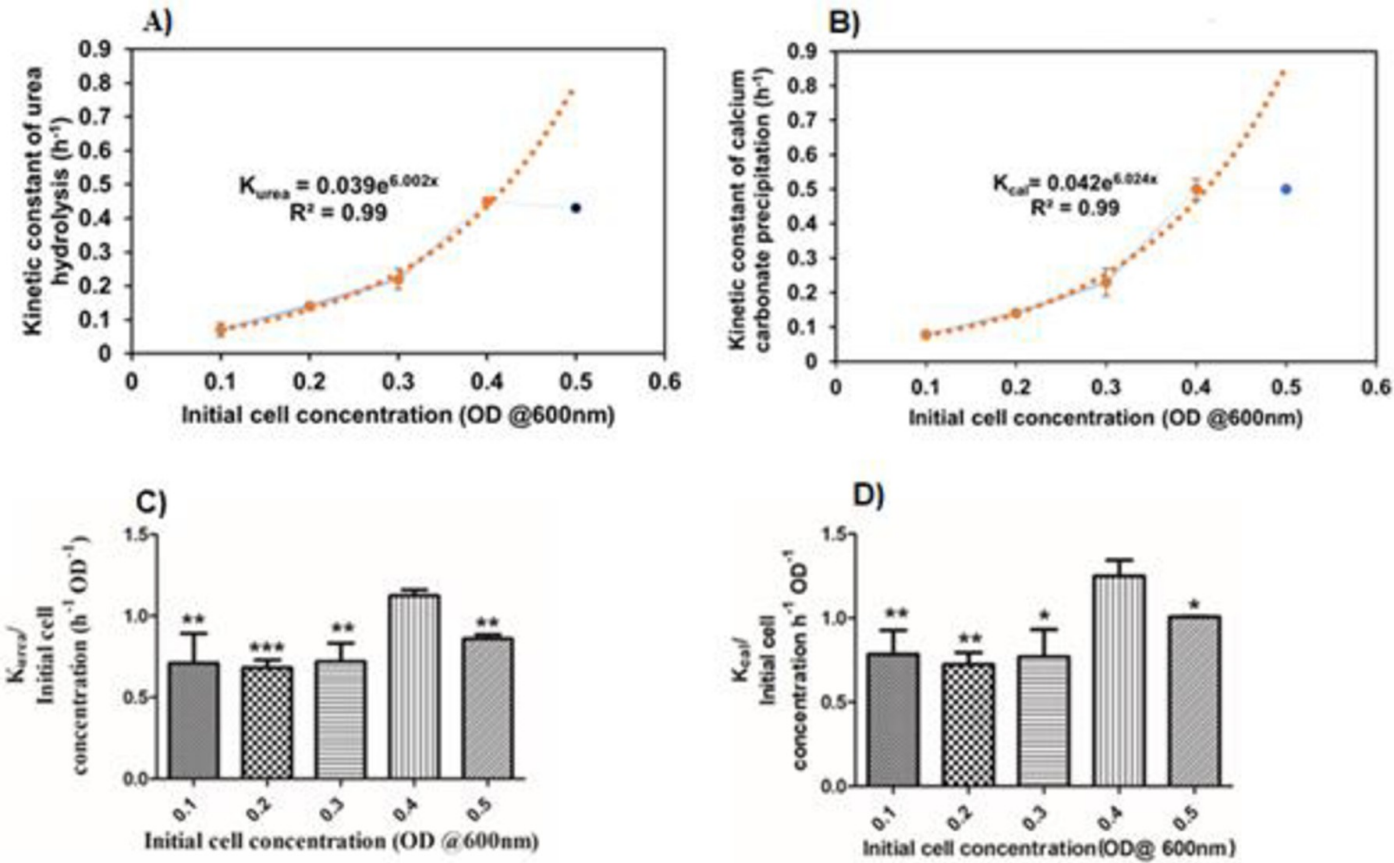
The influence of cell concentration on the urea hydrolysis and CaCO_3_ precipitation. The exponential relationship between kinetic constant of urea hydrolysis (K_urea_) and cell concentration (A), The exponential relationship between kinetic constant of calcium carbonate precipitation (K_cal_) and initial cell concentration (B), The effect of initial cell concentration on the kinetics of urea hydrolysis (C) and calcium carbonate precipitation (D). ** indicates P-value ≤ 0.01, * indicates P-value ≤ 0.05.

Studying the effect of cell concentration on the kinetics of ureolysis is important to understand the process change over time. The kinetic parameters associated with the process can be used to compare the effects of the cell concentration on the process and it is possible to draw the interpretation out of the study. So that, R_urea, Max,_ and K_urea_ values were calculated from Fig 1A (R^2^ values > 0.95). Table 3 shows the calculated values. The lowest and highest value of R_urea, Max_ was found as 25.76 ± 1.12 and 120.47 ± 7.25 for the cell concentration of 0.1 OD and 0.5 OD respectively. Interestingly, the K_urea_ value of 0.4 OD was found to be 0.45 h^-1^ for 0.4 OD of cells which is slightly higher than the K_urea_ value of 0.5 OD of cells: 0.43 h^-1^. Moreover, the K_urea_ values were exponentially increased between 0.1 OD to 0.4 OD (Fig 3A). Eq 5 shows the relationship between K_urea_ and cell concentration. Further, the effect of cell concentration on the kinetics of ureolysis was found by comparing the normalized values of K_urea_ values with its respective cell concentration (Fig 3C) results in the cell concentration has a significant effect on the kinetics of the ureolysis (P-value—0.003). In the end, bacterial cell optical density of 0.4 showed the highest value compared to the other OD values. A student t-test shows the degree of significance mentioned in Fig 3C.

$$K_{urea}\;=\;0.039{\;e}^{6.002x}$$ (5)

K_urea_ = kinetic constant of urea hydrolysis (h^-1^)

x = Initial cell concentration (OD @600 nm) (Between 0.1 to 0.4 OD@600 nm).

**Table 3:** Kinetic parameters associated with urea hydrolysis and CaCO_3_ precipitation.

Initial cell concentration (OD @600nm)	Maximum rate of urea hydrolysis R_urea, Max_ (mM/h)	Kinetic constant of urea hydrolysis K_urea_ (h^-1^)	Maximum rate of Calcium depletion R_cal, Max_ (mM/h)	Kinetic constant of calcium carbonate precipitation K_cal_ (h^-1^)
0.1	25.76 ± 1.12	0.07 ± 0.02	25.96 ± 2.77	0.078 ± 0.01
0.2	43.03 ± 4.85	0.14 ± 0.01	39.76 ± 5.1	0.14 ± 0.01
0.3	63.5 ± 2.7	0.22 ± 0.03	55.75 ± 4.8	0.23 ± 0.04
0.4	99.45 ± 7.9	0.45 ± 0.01	104.01 ± 11.4	0.50 ± 0.03
0.5	120.47 ± 7.25	0.43 ± 0.01	121.4 ± 5.46	0.50 ± 0.01

The R_cal, Max_, K_cal_ are the kinetic parameters calculated from Fig 2A were used to investigate the kinetics of calcium carbonate precipitation (Table 3). Upon increasing the cell concentration, the R_cal, Max_ values also increased. 25.96 mM/h and 121.4 mM/h are the lowest and highest values observed with 0.1 OD and 0.5 OD respectively. The observed K_cal_ values increased exponentially from 0.1 OD to 0.4 OD (Fig 3B). Eq 6 shows the relationship between and K_cal_ and initial cell concentration. 0.5 h^-1^ is the K_cal_ value for both 0.4 OD and 0.5 OD. To understand the effect of initial cell concentration on the calcium carbonate precipitation the K_cal_ values were normalized with their respective cell concentrations and plotted against the cell concentration (Fig 3D). The initial cell concentration has a significant effect on calcium carbonate precipitation (P-value 0.018). A student t-test shows a degree of significance to the other groups comparing 0.4 OD.

$$K_{cal}\;=\;0.042{\;e}^{6.024x}$$ (6)

K_cal_ = kinetic constant of calcium carbonate precipitation (h^-1^)

X = Initial cell concentration (OD @600 nm) (Between 0.1 to 0.4 OD).

### 3.4 Validation of the developed equations

The developed Eqs (5) and (6) have K_urea,_ and K_cal_ as a function of initial cell concentration was validated by performing the same study with 0.25 OD as initial cell concentration (Table 4). It was found that the observed values of kinetic parameters are very close to the predicted values.

**Table 4:** The observed and predicted values of the kinetic parameters using the 0.25 OD as initial cell concentration.

Kinetic parameters	Developed equations	R^2^ values	Observed values	Predicted values
Kinetic constant of urea hydrolysis (h^-1^)	*K*_*urea*_ = 0.039 *e*^6.002*x*^	0.99	0.153 ± 0.01	0.172
Kinetic constant of calcium carbonate precipitation (h^-1^)	*K*_*cal*_ = 0.042 *e*^6.024*x*^	0.99	0.182 ± 0.04	0.190

K_urea_-Kinetic constant of urea hydrolysis, K_cal_-Kinetic constant of CaCO_3_ precipitation. The boundary conditions of the developed equations are 0.1 OD to 0.4 OD initial cell concentration.

### 3.5 Effect of initial cell concentration on the morphology and phase of the CaCO_3_ crystals

**Fig 4:**
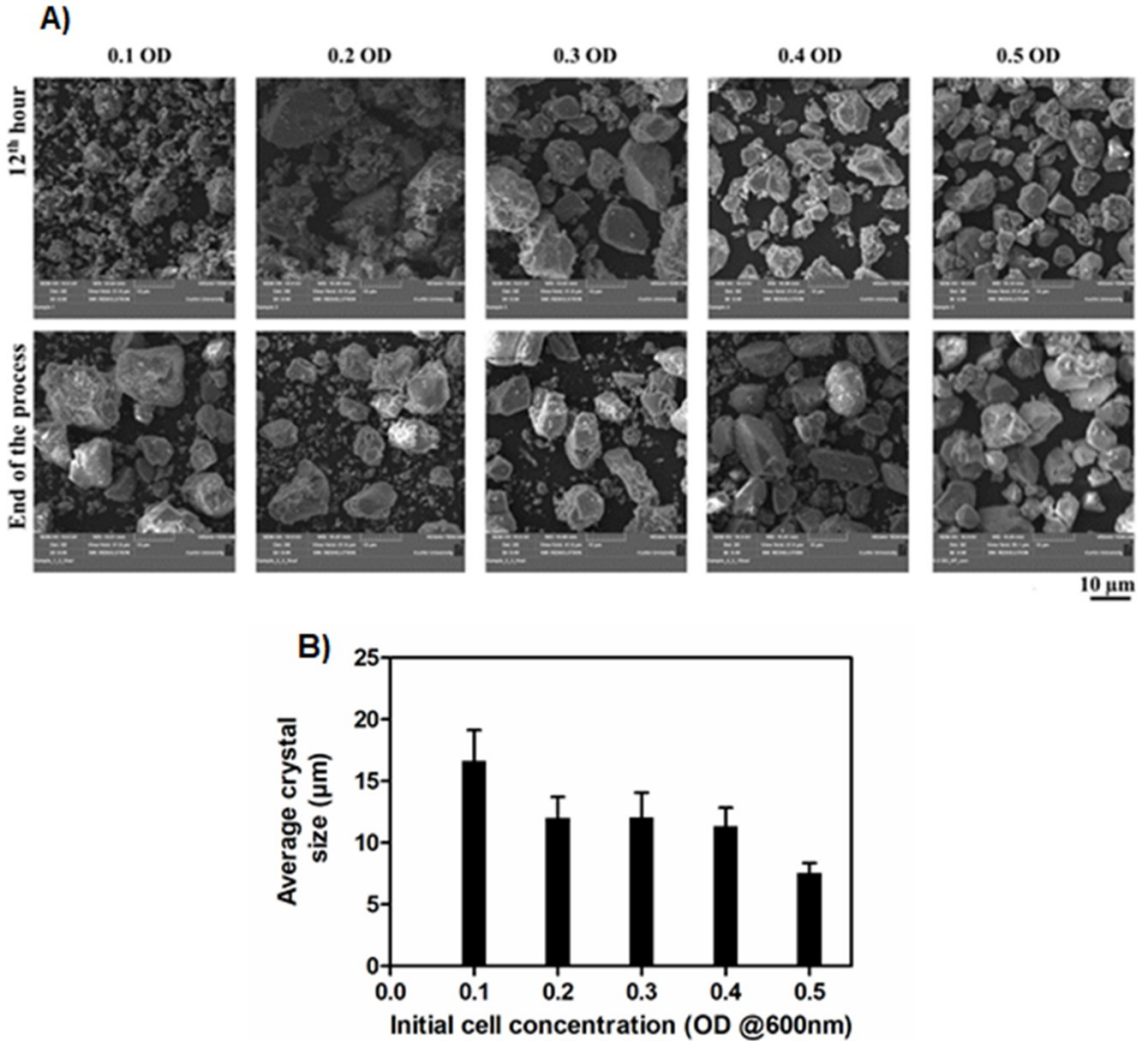
SEM images (A) and sizes (B) of the CaCO_3_ crystals. The SEM images were taken at 12^th^ hour and the end of the process (A). The average size of the CaCO_3_ crystals at the end of the process (B). Crystal sizes were measured using IMAGE J software.

**Fig 5:**
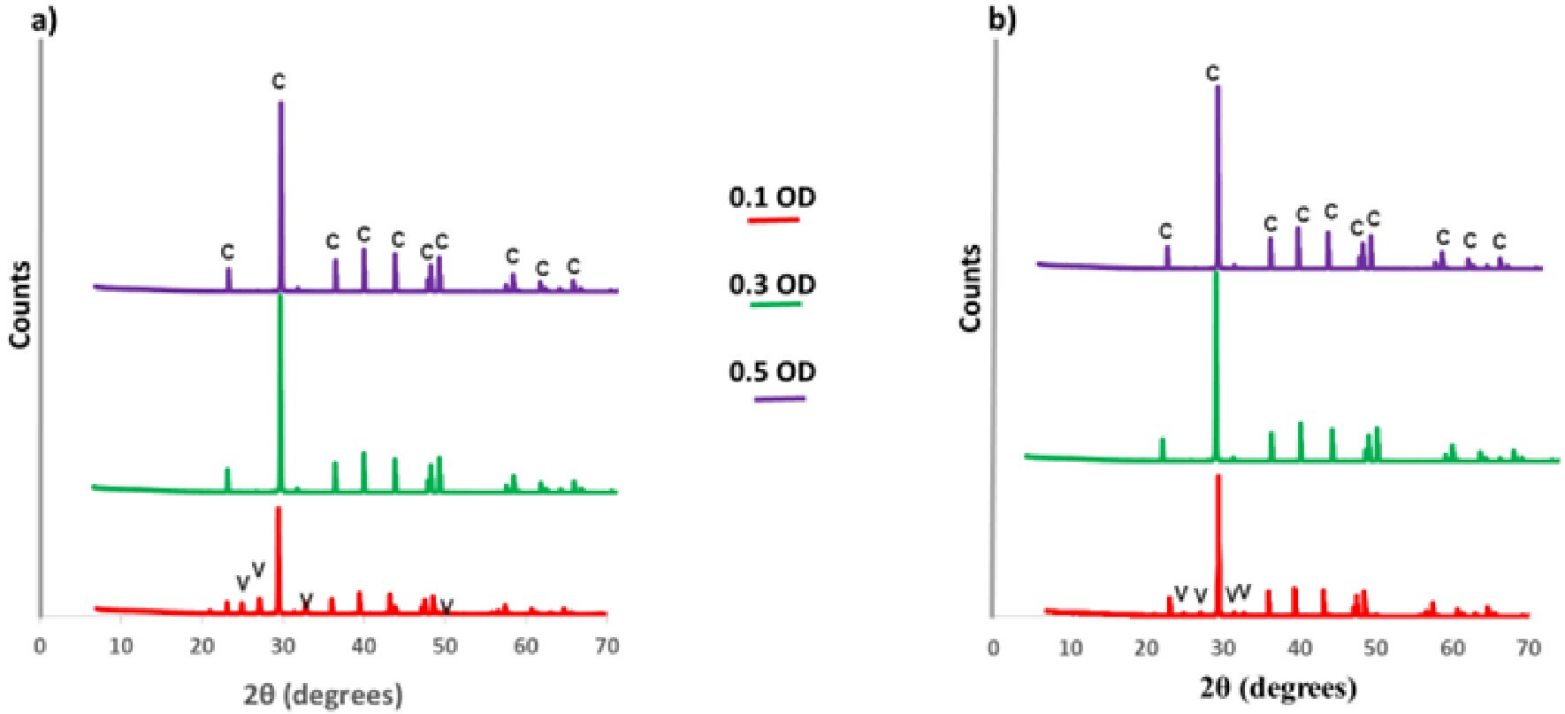
XRD spectrum of CaCO_3_. a) at 12^th^ hour, b) at the end of the process. c-calcite, v-vaterite.

To understand the morphology and phase changes during the precipitation period, size, shape, and phase of the powdered precipitate were studied using the SEM and XRD technique respectively, at the 12^th^ hour and the end of the process (Table 5). Figs 4A and 5 show the SEM images and XRD spectrum of CaCO_3_.

**Table 5:** Morphological and phase observation of calcium carbonate precipitate in medium inoculated with 0.1 OD to 0.5 OD of cells.

Initial cell concentration OD @ 600 nm	Morphology of the crystals at 12^th^ hour of the process				Morphology of the crystals at the end of the process			
	Average size of the crystals (μm)	Shape	Phase		Average size of the crystals (μm)	Shape	Phase	
			% of vaterite	% of calcite			% of vaterite	% of calcite
0.1	7.61±2.1	Polyhedral	33.3	66.7	16.64±2.5	Polyhedral	8.3	91.7
0.2	14.24±2.2		NA	NA	12.01±1.7		0	100
0.3	14.33±2.7		0	100	12.07±2		0	100
0.4	9.31±1.7		NA	NA	11.35±1.5		NA	NA
0.5	7.6±0.9		0	100	7.57±0.8		0	100

NA -Not Analysed.

Considering the size and shape of crystals at the end of the process, SEM images revealed that the size of the crystals decreased when increasing the cell concentration (Fig 4B). The larger and smaller-sized crystals of 16.6 μm and 7.6 μm were formed with cementation medium inoculated with 0.1 OD and 0.5 OD respectively. Polyhedral-shaped crystals are predominantly observed for all the cell concentrations.

The XRD analysis was done to determine the phase composition of the CaCO_3_ crystals at 0.1 OD, 0.3 OD, and 0.5 OD at the 12^th^ hour and the end of the process. In this study, vaterite and calcite polymorphs of CaCO_3_ were recorded. At the 12^th^ hour, the precipitate of 0.1 OD contained 33.3% of vaterite and 66.7% of calcite. But at the end of the process, the composition changed into 8.3% of vaterite and 91.7% of calcite. In the case of 0.3 and 0.5 OD cells, 100% calcite was recorded at both the 12^th^ hour and at the end of the process.

### 3.6 Kinetics of calcium carbonate precipitation after second recharge with and without the addition of fresh bacterial cells

**Fig 6:**
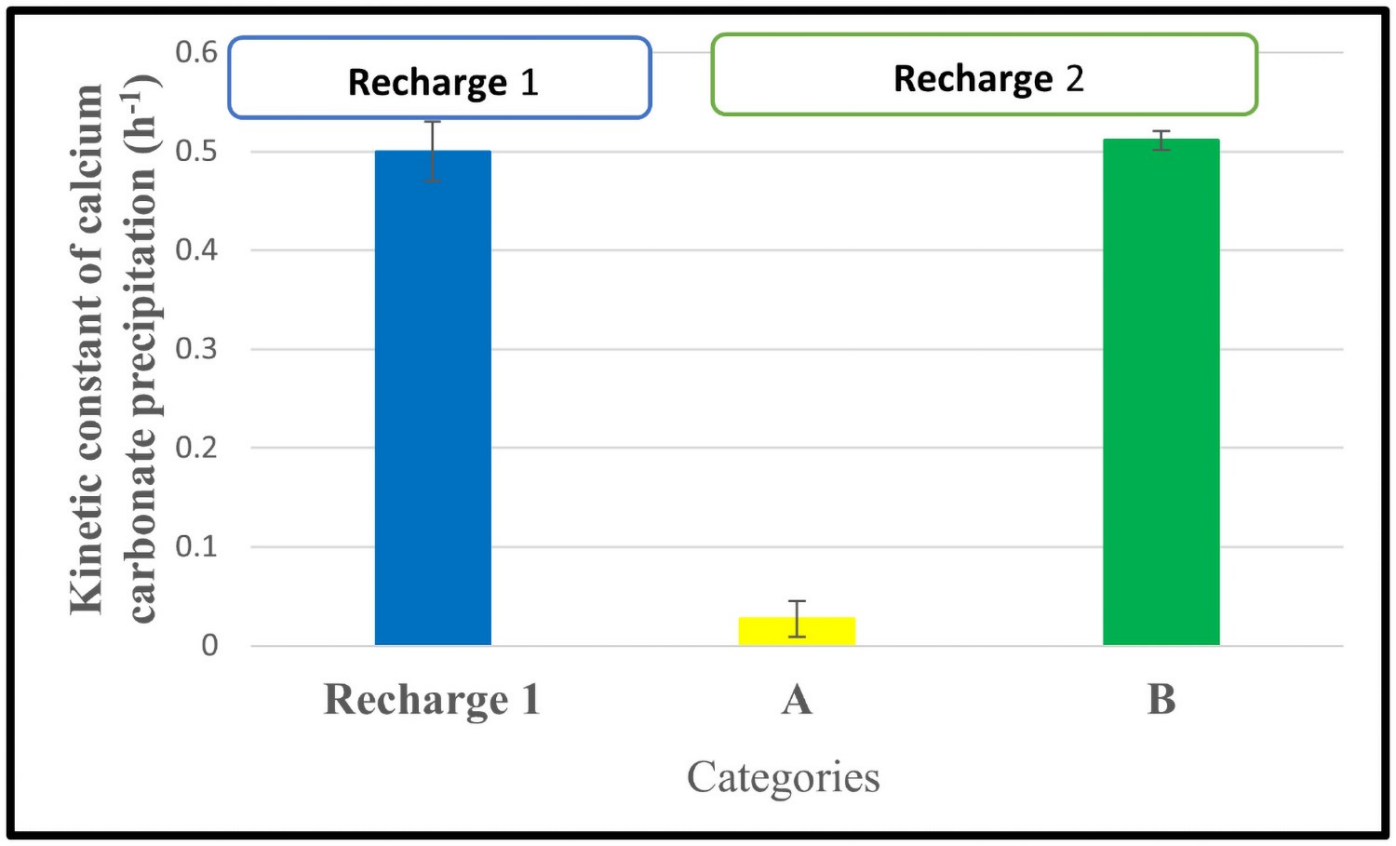
Comparison of kinetic constant values of first and second recharge of calcium carbonate precipitation. A–Set of three flasks used in the 2^nd^ recharge without the addition of bacteria. B–Set of three flasks used in the 2^nd^ recharge without the addition of bacteria.

In this study, the 0.4 OD cell concentration was used as the initial cell concentration because it shows the highest K_cal_ value compared to the other OD values. The calcium profiles of sets A and B were monitored over time until complete precipitation. The monitored profiles were fitted into Eq 4. Fig 6 shows the calculated and compared values of kinetic constant values of CaCO_3_ precipitation.

From Fig 6, in the second recharge, comparing the kinetic constant of calcium carbonate precipitation, set B is higher than set A. The main difference between Set A and Set B is the addition of overnight grown bacteria into set B during the second recharge. It indicates that additional bacteria were required in set A to precipitate CaCO_3_ as quicker as set B.

## 4. Discussion

### 4.1 Effect of cell concentration on the kinetics of urea hydrolysis

To understand the urea hydrolysis at varying initial bacterial cell concentrations of 0.1–0.5 OD, the urea concentration over time in the cementation medium was studied (Fig 1A). The kinetics of ureolysis depend on the bacterial ureolytic activity. The decrease of urea concentration in all the medium indicates that the urea present in the medium was hydrolysed by the bacteria leading to the generation of ammonium ions and pH rise over time (Fig 1A–1C). From the observations, the time required for the active hydrolysis of urea decreased with an increase in the initial cell concentration from 0.1 OD to 0.5 OD. It was reported that neither ammonium ions generation up to 0.19 mM nor pH (within 6 to 9) had a significant effect on the ureolytic ability of the cells [32]. So, the duration required for the active hydrolysis of urea can be minimized by increasing the initial cell concentration. In other terms, a high initial concentration of cells leads to immediate active urea hydrolysis. The influence of cell concentration on urea hydrolysis is further addressed in detail by recording the kinetic parameters associated with the process (Table 3). Most importantly, the observed exponential relationship (Eq 5) between the K_urea_ and initial cell concentration between 0.1 OD to 0.4 OD indicates the rate of urea hydrolysis can be improved by increasing the initial cell concentration. It is mainly due to the positive relationship between the number of bacterial cells and the total urease production in the system [12]. A similar positive correlation was reported between the urea hydrolysis rate and cell concentration [30, 32]. Furthermore, the developed Eq 5, between K_urea_ and cell concentration is validated by using 0.25 OD cell concentration (Table 4). It can be incorporated into rate predictions and the optimization of ureolytic MICP applications between 0.1 OD to 0.4 OD cell concentration.

In Fig 3C, 0.4 OD of bacteria shows a significantly higher value of K_urea_/OD than other OD values. This result shows that 0.4 OD is the initial cell concentration at which the rate of urea hydrolysis is better. Further, the result shows that 0.4 OD of initial cell concentration has performed even better than 0.5 OD of initial cell concentration (P-value 0.027). It is mainly due to the rate of urea hydrolysis which affected the precipitation process. The precipitation on the bacterial surface limits the nutrient transport including urea across the cell membrane or inactivation of the cells [5, 6, 37]. So, there is the critical initial cell concentration at which the rate of urea hydrolysis is maximum. From these results, it is evident that the initial concentration of the bacteria needs to be optimized based on the conditions employed in the process.

### 4.2 Effect of cell concentration on the kinetics of CaCO_3_ precipitation

The kinetics of calcium carbonate precipitation majorly depends on the rate of urea hydrolysis by the bacteria. The inverse relationship between the concentration of soluble calcium ions and insoluble calcium carbonate precipitate indicates that the soluble calcium ions are converted into the insoluble calcium carbonate precipitate (Fig 2A and 2B). In the initial phase of CaCO_3_ precipitation, the minimum rate of CaCO_3_ with lower cell concentration denoted that lower cell concentration needs a certain time to adapt to the cementation environment. In the middle phase, the highest value of the R_cal, Max_ was observed to be 121.4 ± 5.6 mM/h (Table 3) at 0.5 OD initial cell concentration due to the R_urea, Max_ which is equivalent to the maximum productivity of 12.1 g L^-1^ h^-1^ of CaCO_3._ In the later phase, the rate of CaCO_3_ precipitation decreased due to the decrease in ureolytic activity of cells caused by the encapsulation of CaCO_3_ precipitate on the cell surface [37].

The k_cal_ increased exponentially (Fig 3B) between 0.1 OD to 0.4 OD of initial cell concentration. The minimum k_cal_ value of 0.079 ± 0.014 h^-1^ was observed with inoculated with 0.1 OD and the maximum was noted as of 0.5 ± 0.03 and 0.5 ± 0.01 h^-1^ with both 0.4 OD and 0.5 OD as initial cell concentration respectively. The maximum values of kinetic constants in 0.4 OD and 0.5 OD is mainly due to the higher surface area available for the binding of calcium ions that results in more available nucleation sites for calcium carbonate precipitation [16] and the faster rate of ${CO}_{3}^{2-}$ ions generation due to the ureolytic activity of cells in the given system [38].

The k_cal_ normalized with initial cell concentration was plotted to determine the effect of initial concentration on the kinetics of calcium carbonate precipitation (Fig 3D). The medium having 0.4 OD of initial cell concentration has shown the maximum value of K_cal_/ initial cell concentration of 1.25 ± 0.09 h^-1^ OD^-1^. This value is significantly higher than the medium having 0.5 OD as the initial cell concentration. It is mainly due to the CaCO_3_ precipitation on the bacterial surface that limits the CaCO_3_ precipitation potential of the bacteria [3, 37]. Hence, it is noteworthy, to achieve the maximum rate of CaCO_3_ precipitation the initial concentration of the cells needs to be fixed for the particular concentration of urea and calcium (In this study we used 0.5 M urea and calcium).

From the kinetic constant values in Table 3, the rate of calcium carbonate precipitation is highly associated with the rate of urea hydrolysis. It indicates that urea hydrolysis and CaCO_3_ precipitation happens in the system simultaneously. The concentration of urea, ammonium ions, pH, and soluble and insoluble concentration of calcium ions can be the parameters to monitor the calcium carbonate precipitation process. The notable kinetic parameters associated with this study were compared with previous literature and tabulated (Table 6).

**Table 6:** Comparison of kinetic constants reported in the previous literature with the current study.

Parameter	Current study	[30]	[32]	[12]
R_urea, Max_ (mM/h)	120.47 ± 7.25	NR	124 ± 13	NR
R_cal, Max_ (mM/h)	121.4 ± 5.6	NR	NR	NR
Maximum value of K_urea_ (h^-1^)	0.45 ± 0.014	0.039	0.38	NR
Maximum value of K_cal_ (h^-1^)	0.5 ± 0.01	NR	NR	0.048

NR–Not reported. R_urea, Max_–Maximum rate of urea hydrolysis, R_cal, Max_–Maximum rate of CaCO_3_ precipitation, K_urea_–Kinetic constant of urea hydrolysis, K_cal_–Kinetic constant of CaCO_3_ precipitation.

### 4.3 Influence of cell concentration on morphology and phase of CaCO_3_ crystals

The bacteria act as a nucleation site for the CaCO_3_ precipitation and it can influence the size and shape of the CaCO_3_ crystals [26, 27]. The CaCO_3_ crystal morphology depends on the various parameters including the concentration of urea, calcium, and nutrients present in the cementation medium and extracellular polymeric substances present on the cell surface [39, 40]. In Fig 4B at the end of the process, 0.1 OD shows bigger-sized crystals than other OD values because the number of bacteria is proportional to the available nucleation sites for the CaCO_3_ precipitation. It clearly says that the lesser the availability of nucleation sites the size of the crystal will be higher [16]. From Table 5, the medium with other than 0.1 OD reaches its saturated size around the 12^th^ hour after that size of the crystals not increased. It reveals the kinetics associated with a cell number in the cementation medium determines how fast crystals should grow to reach their saturated size in the given system.

The shape and size of the crystals can be influenced by the bacterial size, shape, and assembly of CaCO_3_ on the cell surface [40]. The observed polyhedral shape crystals in this study (Fig 4A) are due to the formation of calcite polymorph crystals. From the qualitative EDS results, it was clear that the precipitate is composed of only calcium, carbon, and oxygen which forms calcium carbonate precipitate (S1 Fig).

As the CaCO_3_ precipitation progresses, the amorphous CaCO_3_ undergoes a phase transition to metastable vaterite and then to a more stable calcite with respect to the supersaturation of the system [40]. The organics such as extracellular polymeric substances that surround the bacterial surface and acidic amino acids synthesized by the bacteria are the major factors that stabilize metastable vaterite phase formation [12, 41]. From Table 5, in the case of 0.1 OD cell concentration, the calcite composition at the end of the process increased from 66.7% (at the 12^th^ hour) to 91.7%. This result shows that the transformation of polymorph occurred from vaterite to calcite and it could be explained by the Ostwald rule of phase transitional theory. Polymorph transition occurs from thermodynamically metastable vaterite to more stable calcite [42–44]. Whereas in the case of 0.3 and 0.5 OD cell concentration only the calcite polymorph of crystals was present at the 12^th^ hour and the end of the process. It indicates that the polymorph composition of CaCO_3_ depends on the kinetics of CaCO_3_ precipitation influenced by the initial cell concentration. The kinetics of ureolysis has a direct influence on the rate of ammonium ions and hydroxide ions generation. The fast rate of ammonium and hydroxide ions generation alters the solution chemistry of the cementation medium quickly. In the case of 0.3 and 0.5 OD, quick alteration in the cementation medium could lead to faster formation of calcite crystals whereas no vaterite crystals were observed at the 12^th^-hour. The pH change throughout the process indicates that the solution chemistry change. Towards the alkaline pH, it favours calcite formation of crystals than other polymorphs [22, 45]. The most stable polymorph of calcium carbonate (calcite) was observed in this study. Hence the process could be considered for various engineering applications [25].

### 4.4 Kinetics of calcium carbonate precipitation after second recharge with and without addition of bacterial cells

The large-scale application of MICP in soil improvement needs multiple recharge of cementation medium [6, 8]. In that case, understanding the changes in the rate of CaCO_3_ precipitation due to the cell concentration after the subsequent recharge of the cementation medium is important to design the successful process. In this study, our results show (Fig 6) the K_cal_ value decreased from 0.5 h^-1^ to 0.048 h^-1^ (Set A) after adding the cementation medium in the second recharge. On the other hand, the addition of bacteria along with cementation medium during the second recharge (Set B) shows an almost similar K_cal_ value to the first recharge. These results indicate that the available cell concentration in the cementation medium at the end of the first recharge is not enough to keep the first recharge rate of CaCO_3_ precipitation when it goes into the subsequent recharges. This basic information needs to be considered when designing the MICP for large-scale applications.

## 5. Conclusions

We studied the influence of cell concentration on the efficacy of microbially induced CaCO_3_ precipitation. We observed that the initial cell concentration directly affects the kinetics of calcium carbonate precipitation and quality of the precipitate. The major conclusions of the study are:

1. High rate of CaCO_3_ precipitation occurs at high cell concentrations. In this study, the medium inoculated with 0.4 OD has shown maximum performance compared to other cell concentrations with the K_cal_ value of 0.5 h^-1^ and complete conversion into insoluble calcium carbonate occurred in around 6 hours.
2. The performance of 0.4 OD was significantly higher than 0.5 OD (Fig 3C and 3D) indicating that the optimum initial cell concentration needs to be fixed based on the application and initial concentration of cementation media.
3. The kinetics of calcium carbonate precipitation have a positive relationship with initial cell concentration (0.1 OD to 0.4 OD). Our developed Eq 6 can be incorporated into the kinetic model of CaCO_3_ precipitation for soil stabilization applications (for cell concentration between 0.1 OD to 0.4 OD in the given cementation medium and conditions).
4. The kinetics of the process have a direct influence on calcium carbonate crystal morphology and phase. The slower the kinetics of the CaCO_3_ precipitation, the higher is the size of the crystal. Also, the kinetics of CaCO_3_ precipitation positively influence the crystal phase transformation. The faster the kinetics, the quicker is the transformation of vaterite to calcite.
5. We have observed that thermodynamically stable calcite polymorph phase is predominant after completion of precipitation process in all the treatments. So, the cell concentration employed in this study is useful for different engineering applications.
6. From the multiple recharge study, the addition of bacteria along with cementation medium during subsequent recharge is essential for successful completion of the process at a faster rate.

## 6. Future directions

For the next phase of this research, different bacterial strains and native communities can be employed to investigate their performance under field conditions. The kinetic parameters derived from the current study will be useful to design the application process for the next phase. It is imperative to understand the influence of initial cell concentration on nanomechanical properties of microbially induced carbonate crystals in details. More tests need to be done to widen the scope of current studies with different metabolic pathways as denitrification, sulphate reduction, carbonic anhydrase to eliminate the effects of ammonia generated in the ureolytic pathway of MICP as utilised in the current study.

## Supporting information

S1 Fig EDS spectrum of CaCO_3_ precipitate in medium inoculated with 0.1 OD. Similar spectrum was observed with other initial cell concentrations (0.2–0.5 OD). cps/ev–counts per second per electron volt, keV–kilo electron Volts.
(DOCX)
