# Supplementary material for: Insights into the influence of cell concentration in design and development of microbially induced calcium carbonate precipitation (MICP) process
Source: PLoS One. 2021 Jul 12;16(7):e0254536. doi: 10.1371/journal.pone.0254536 (PMC8274927; doi:10.1371/journal.pone.0254536)
Supplement: S1 Fig — Similar spectrum was observed with other initial cell concentrations (0.2–0.5 OD). cps/ev–counts per second per electron volt, keV–kilo electron Volts. (DOCX) [file pone.0254536.s001.docx]

**.**


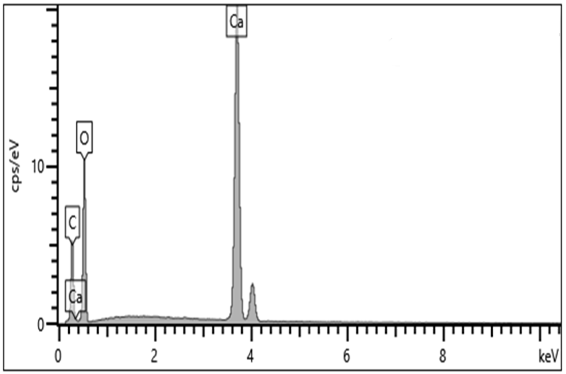


**S1 Fig. EDS spectrum of CaCO_3_ precipitate in medium inoculated with 0.1 OD (Similar spectrum was observed with other initial cell concentrations (0.2-0.5 OD). cps/ev – counts per second per electron volt, keV – kilo electron Volts.**
